# Oxidative Stress Related to Plasmalemmal and Mitochondrial Phosphate Transporters in Vascular Calcification

**DOI:** 10.3390/antiox11030494

**Published:** 2022-03-02

**Authors:** Nhung Thi Nguyen, Tuyet Thi Nguyen, Kyu-Sang Park

**Affiliations:** 1 Department of Physiology, Wonju College of Medicine, Yonsei University, Wonju 26426, Korea; nhung.nt@vinuni.edu.vn; 2 Mitohormesis Research Center, Wonju College of Medicine, Yonsei University, Wonju 26426, Korea; 3 Medical Doctor Program, College of Health Sciences, VinUniversity, Hanoi 12406, Vietnam; 4 Internal Medicine Residency Program, College of Health Sciences, VinUniversity, Hanoi 12406, Vietnam

**Keywords:** vascular calcification, oxidative stress, phosphate, calcium, mitochondria, transporters

## Abstract

Inorganic phosphate (Pi) is essential for maintaining cellular function but excess of Pi leads to serious complications, including vascular calcification. Accumulating evidence suggests that oxidative stress contributes to the pathogenic progression of calcific changes. However, the molecular mechanism underlying Pi-induced reactive oxygen species (ROS) generation and its detrimental consequences remain unclear. Type III Na^+^-dependent Pi cotransporter, PiT-1/-2, play a significant role in Pi uptake of vascular smooth muscle cells. Pi influx via PiT-1/-2 increases the abundance of PiT-1/-2 and depolarization-activated Ca^2+^ entry due to its electrogenic properties, which may lead to Ca^2+^ and Pi overload and oxidative stress. At least four mitochondrial Pi transporters are suggested, among which the phosphate carrier (PiC) is known to be mainly involved in mitochondrial Pi uptake. Pi transport via PiC may induce hyperpolarization and superoxide generation, which may lead to mitochondrial dysfunction and endoplasmic reticulum stress, together with generation of cytosolic ROS. Increase in net influx of Ca^2+^ and Pi and their accumulation in the cytosol and mitochondrial matrix synergistically increases oxidative stress and osteogenic differentiation, which could be prevented by suppressing either Ca^2+^ or Pi overload. Therapeutic strategies targeting plasmalemmal and mitochondrial Pi transports can protect against Pi-induced oxidative stress and vascular calcification.

## 1. Introduction

Vascular calcification includes intimal and medial calcification, which occur via two different pathological mechanisms. Intimal calcification involves the formation of atherosclerosis plaques with necrotic lipid core and activation of macrophages, which leads to endothelial cell inflammation. Blood flow is obstructed when the plaques rupture, causing ischemia. Medial calcification is an active and complex cell-mediated process characterized by the absence of inflammatory cells and is associated with inorganic phosphate (Pi) and calcium overload in vascular smooth muscle cells (VSMCs). It is also known as Mönckeberg’s sclerosis and causes arterial stiffness and decreases the compliance of vessels, which is observed in aging individuals and those with chronic kidney disease and diabetes mellitus. Medial calcification can happen in the small arteries of the toes to large arteries such as the tibial, popliteal, and femoral arteries of the legs or the carotid arteries [1,2,3].

Research shows that several key events mediated by Ca^2+^ and Pi occur simultaneously in VSMCs. Under hyper-phosphatemic conditions, VSMCs are trans-differentiated into bone-like cells that express high levels of osteogenic markers, including Runt-related transcription factor 2 (Runx2), osteopontin (OPN), osteocalcin, alkaline phosphatase (ALP), SRY-Box transcription factor 9 (Sox9), and type II and X collagen (Col II and Col X), and low levels of α-smooth muscle actin (αSMA) and smooth muscle 22α (SM22α). Osteogenic trans-differentiation can be associated with other phenomena, including loss of inhibitors such as matrix Gla protein, pyrophosphate, and fetuin A, or enhancement of cell senescence features, such as DNA damage and oxidative stress.

So far, studies have described the pathogenesis of Pi-induced calcification related to osteogenic differentiation and apoptotic body formation in VSMCs. Some of them have emphasized the role of cytosolic and mitochondrial oxidative stress upstream of the upregulation of osteogenic genes and changes in calcification; however, the molecular mechanisms connecting high Pi levels with oxidative stress have not been elucidated. To understand the pathogenic processes underlying cellular Pi overload, research on how Pi enters the cytosol and is taken up by the mitochondrial matrix must be conducted.

The estimation of cytosolic and mitochondrial Pi concentration using ^31^P NMR and LC/MS/MS has been reported [4]. In insulin-secreting cells, 2.8 nmol Pi/mg protein was found in the cytosol, while the mitochondrial Pi concentration (10 nmol/mg protein) was much higher. Based on rough calculations using the cell volume and volume proportion of the mitochondrial fraction, the cytosolic and mitochondrial Pi concentrations are approximately 1.2 mM and 9–12 mM, respectively. Considering that the normal plasma Pi level is in the range of 1.12–1.45 mM, the Na^+^ gradient across the plasma membrane might be enough to drive cellular Pi uptake through an Na^+^-Pi cotransport mechanism. However, Pi transport into the mitochondrial matrix has a steep uphill gradient, which could be closely affected by mitochondrial activities, but details about the transportation mechanism remain unclear. In this review, we introduced the pathophysiological characteristics of plasmalemmal Pi transporters and mitochondrial Pi carriers in vascular calcification, particularly focusing on the vicious cycle between Pi uptake and oxidative stress.

## 2. Plasmalemmal Phosphate Transporters

**Figure 1:**
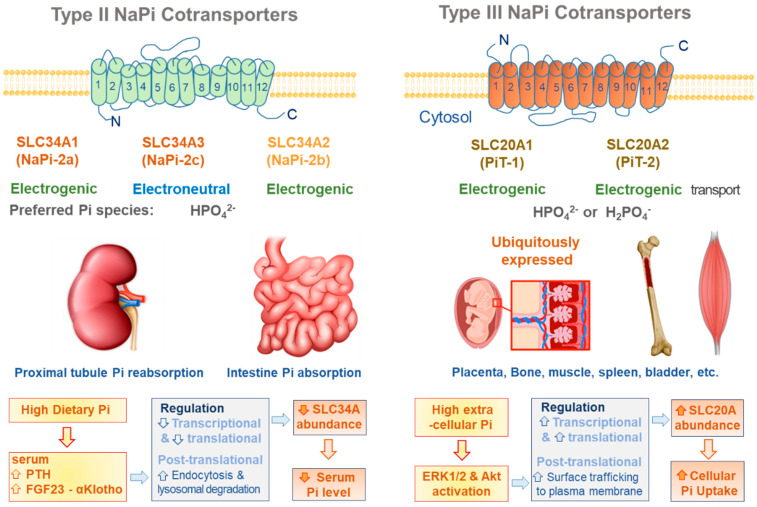
Plasmalemmal Pi transporters. The characteristics and tissue expressions of type II and III NaPi cotransporters are illustrated. The functional abundance of type II NaPi cotransporters are regulated by hormones, such as PTH and FGF23, in order to normalize serum Pi levels upon the alterations in dietary Pi. However, high extracellular Pi directly increases the functional abundance of type III NaPi cotransporters, leading to intracellular Pi accumulation.

Mainly two solute carrier families, SLC34A and SLC20A, transport extracellular Pi across the plasma membrane (Figure 1), both of which use the chemical gradient of Na^+^ to transport Pi [5,6,7]. The SLC34A family has three members, including sodium-phosphate cotransporter types IIa (NaPi-IIa, SLC34A1) and IIc (NaPiIIc, SLC34A3), which are mainly expressed in the brush border membrane of proximal tubules, and type IIb (NaPi-IIb, SLC34A2), which is primarily expressed in the gastrointestinal tract [5,6,7,8,9]. The SLC20A family consists of two members, PiT-1 (SLC20A1) and PiT-2 (SLC20A2), which are ubiquitously expressed in the human body [5,6,7,9]. The physiological role of the SLC34 family proteins in Pi transport has been extensively studied and well characterized, including in dietary Pi absorption in small intestinal epithelial cells and renal Pi reabsorption in proximal tubular cells [7,10,11,12,13,14]. The SLC20A family has been considered to play a housekeeping role in Pi homeostasis in various tissues [8]. However, recent studies have shown their pathological roles in vascular calcification [15,16,17] and Pi-induced apoptosis in endothelial cells, epithelial cells, and podocytes [18]; hence, they are suggested to be putative therapeutic targets for vascular calcification.

### 2.1. SLC34A Family

All members of the SLC34 family transport HPO_4_^2−^ preferentially with Na^+^, although the stoichiometry between Na^+^ and Pi differs among the three members. A series of studies by Forster’s group using the patch-clamp technique in *Xenopus laevis* oocytes showed that NaPi-IIa/NaPi-IIb are electrogenic and that NaPi-IIc is electroneutral with Na:Pi stoichiometry of 3:1 and 2:1, respectively [5,6,8,9,14,19]. Transport of Na^+^ and Pi via SLC34 is affected by extracellular pH, which influences the distribution of H_2_PO_4_^−^ or HPO_4_^2−^ [6,8,19]. This family mostly consists of functional monomers, with some occasional dimers or tetramers [6,8]. The members of this family are composed of 599 to 640 amino acids [6,19] with 12 transmembrane domains. Both the C- and N-termini are intracellular [6], while the linker region is located on the extracellular side. NaPi-IIa and NaPi-IIc, expressed abundantly in the apical membrane of proximal tubules, play essential roles in Pi reabsorption.

A previous study has shown that mice lacking NaPi-IIa had low plasma Pi level with high urinary Pi excretion. In contrast, mice lacking NaPi-IIc did not show significant hypophosphatemia [9]. However, double knockout of both NaPi-IIa and NaPi-IIc resulted in serious renal Pi wasting, hypophosphatemia, and impaired skeletal mineralization, which are more severe than the phenotypes observed after knocking out only NaPi-IIa. In humans, loss of function mutations in *SLC34A1* and *SLC34A3* cause hereditary hypophosphatemia, accompanied by increase in urinary loss of Pi [20]. NaPi-IIb expressed in the small intestine, regulates Pi absorption from the daily diet [21,22]. NaPi-IIb^−/−^ mice died at the embryonic stage, whereas heterozygous mice showed lower plasma Pi level at 4 weeks. At 20 weeks, these heterozygous mice returned to normophosphatemia with increase in the abundance of NaPi-IIa and NaPi-IIc [22]. This indicated that Pi absorbed via NaPi-IIb is critical for survival during embryogenesis; however, compensatory upregulation of other types of NaPi transporters occur after birth.

### 2.2. SLC20A Family

The SLC20A family consists of two members, PiT-1 and PiT-2. These isoforms were initially discovered as retroviral receptor Glvr-1 (gibbon ape leukemia virus receptor) and Ram-1 (rat amphotropic leukemia virus receptors) [23,24], and then later identified to be sodium-dependent phosphate transporters [25,26]. An electrophysiologic study in *Xenopus* oocytes has shown that Pi transport via SLC20A is electrogenic with Na^+^:Pi stoichiometry of 2:1, which is negligibly sensitive to pH and phosphonoformic acid (PFA) [8]. H_2_PO_4_^−^ was suggested as the preferential form of Pi for transport via PiT-1/2 [8]. In HEK293 cells overexpressing PiT-1, extracellular Pi elicited inwardly rectifying currents, possibly leading to plasmalemmal depolarization. Pi-induced inward currents were exclusively extracellular and Na^+^-dependent, as indicated by increase in Na^+^ uptake after addition of extracellular Pi [27]. However, in this study, Pi-induced inward currents were proportionally decreased by extracellular acidification (pH 6.6), because of which more H_2_PO_4_^−^ is produced from HPO_4_^2−^ [27]. These results implied that H_2_PO_4_^−^ is more favored than other Pi species in Pi-transport via PiT-1, which, however, requires further clarification. PiT-1/2 has 12 transmembrane domains with both the C- and N-termini facing the extracellular space [8].

Mice lacking PiT-1 are embryonic lethal due to impaired hemopoiesis in the liver [28]. Interestingly, loss of function mutation in human *SLC20A2* was responsible for basal ganglia calcification. The ectopic calcification can be explained by the high levels of extracellular Pi arising due to impaired cellular uptake via PiT-2, leading to the accumulation of calcium and Pi aggregates [29]. Consistently, *SLC20A2* knockout mice also showed abnormal calcifications in the thalamus, basal ganglia, and cerebral cortex with maintained high Pi levels in cerebrospinal fluid (CSF) [29,30]. These findings suggested an essential role of PiT-2 in cellular Pi uptake for the maintenance of physiological Pi concentration in the extracellular environment.

Critical pathogenic roles of PiT-1/2 in vascular calcification have been demonstrated. In human VSMCs, shRNA-mediated knockdown of PiT-1 suppressed Pi-induced change in osteochondrogenic phenotype and vascular calcification. Similar observations were made in osteoblasts where PiT-1 knockdown reduced matrix mineralization and the expression of osteoblast markers such as OPN. As a compensatory change, the transcriptional level of PiT-2 increased two-fold in PiT-1-deleted VSMCs [31]. Silencing of either PiT-1 or PiT-2 protected from Pi-induced cytotoxicity in insulin-secreting cells, although PiT-1 suppression showed slightly more inhibition than PiT-2 silencing. Knockdown of both PiT-1 and PiT-2 successfully prevented mitochondrial superoxide generation, permeability transition (PT) pore opening, endoplasmic reticulum (ER) stress, and defective insulin secretion induced by the presence of high levels of Pi [27]. Double knockdown of PiT-1 and PiT-2 also abolished intracellular calcification by high Pi levels in rat aortic smooth muscle cells [32]. However, it has recently been reported that PiT-2 haploinsufficiency enhanced the development of vascular calcification, suggesting the protective role of PiT-2 against Pi-induced calcification [33]. Further investigations are required to clarify the pathophysiologic difference between PiT-1 and PiT-2 in VSMCs.

### 2.3. Cytosolic Phosphate Exporter

Unlike SLC34A and SLC20A, which mediate cellular Na^+^ and Pi uptake, xenotropic and polytropic retrovirus receptor 1 (XPR1) was identified as a Na^+^-independent Pi exporter in mammalian cells, which was initially known as a receptor for murine leukemic viruses [34]. XPR1 contains the SPX domain in the N-terminus, which is involved in Pi sensing and transport [35]. Functional expression of XPR1 has been reported in brain tissues and neural stem cells, and an inactivating mutation in XPR1 results in basal ganglia calcification due to intra-neuronal Pi deposition [36]. Intriguingly, this phenotype is similar to that of *Slc20a2* deletion, which induces extra-neuronal Pi accumulation as described above. A recent study has demonstrated the functional role of XPR1 in renal Pi reabsorption [37], although its transport and regulatory mechanisms remain unclear.

### 2.4. Oxidative Stress Related to Plasmalemmal Pi Transporters

Pi uptake with Na^+^ via PiT-1 is an electrogenic process, resulting in net inward currents. PiT-1/2-mediated transport (symport) can depolarize plasma membrane potential, leading to the opening of voltage-gated calcium channels (VGCCs) highly expressed in VSMCs. Park’s group reported that high Pi elicited dose-dependent plasmalemmal depolarization in rat aortic smooth muscle cells, which was prevented by silencing PiT-1/2 [22,37]. Depolarization of VSMCs elicited VGCC-mediated increase in cytosolic Ca^2+^ concentration ([Ca^2+^]_i_), which was dependent on extracellular Ca^2+^ concentration and was blocked by VGCC inhibitors, verapamil or nimodipine. Furthermore, Pi-induced elevation in [Ca^2+^]_i_ was abolished in cells lacking PiT-1/2 or by extracellular Na^+^ free condition [22]. These findings indicated that cellular Pi uptake via PiT-1/2 leads to Ca^2+^ influx due to alteration of plasma membrane potential, consequently resulting in accumulation of Ca^2+^ and Pi in the cytosol. Both Ca^2+^ and Pi elevation accelerated reactive oxygen species (ROS) generation and vascular calcification, all of which were prevented by VGCC inhibitors or intracellular Ca^2+^ chelation [22].

The importance of PiT-1/2 in vascular calcification is evident from the increase in the expression level of PiT-1/2 in high Pi environment. In insulin-secreting cells and aortic smooth muscle cells, Pi increased the transcriptional and translational levels of PiT-1 and PiT-2 [27,32]. In addition, acute exposure to high Pi (for <1 h) facilitated the trafficking of PiT-1 to the plasma membrane, increasing functional Pi transporters for cellular Pi uptake. PiT-1/2, upregulated by Pi induction, may engage in a feed-forward amplification loop to accelerate pathogenic Pi overload in the cytosol.

Cellular Pi uptake via PiT-1/2 activates ERK1/2 and mTOR signaling and its downstream p70S6K in VSMCs and insulin-secreting cells [32]. Subsequently, activation of ERK1/2 and mTOR signaling participates in Pi-induced PiT-1/2 upregulation. Consistently, high Pi-induced oxidative stress via PiT-1/2 critically relies on ERK1/2-mTOR signaling. The molecular mechanism linking ERK1/2-mTOR signaling and calcification has not yet been elucidated; however, the translocation of NF-κB into the nucleus and upregulation of osteogenic genes, including *RUNX2* and *OPN* were abolished by the blockade of either ERK1/2 or mTOR [32]. Taken together, PiT-1/2-mediated Pi uptake and the consequent activation of the ERK1/2-mTOR axis play an essential role in the vicious cycle of oxidative stress and vascular calcification.

## 3. Mitochondrial Phosphate Transporters

**Figure 2:**
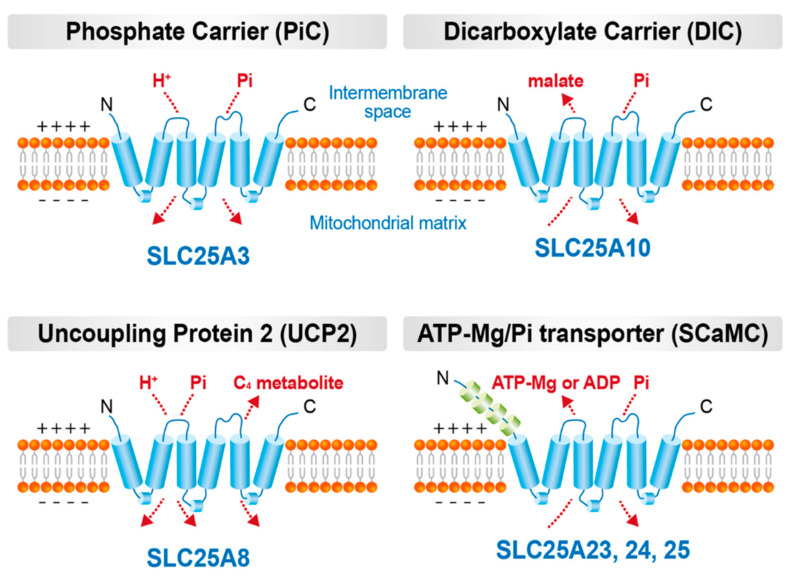
Mitochondrial Pi transporters. Four mitochondrial carriers involved in Pi transport across the inner mitochondrial membrane and their hypothetical transport mechanisms are illustrated. PiC is the main route of mitochondrial Pi uptake driven by pH gradient. DIC exchanges dicarboxylate for Pi or other dicarboxylate, but also transport adenine nucleotides and glutathione. UCP2 performs Pi transport with C_4_ metabolites including malate, oxaloacetate, and aspartate. ATP-Mg/Pi transporter has EF hand domains in N-terminal leading to having a Ca^2+^ activated properties.

Pi acts as an essential molecule for many cellular functions. After entering the cytosol, Pi participates in cellular signaling such as those involving phosphorylation. In the mitochondria, Pi is used by ATP synthase to phosphorylate ADP after it enters the matrix via adenine nucleotide translocase (ANT). Furthermore, other than acting as a substrate for ATP synthesis, Pi also activates mitochondrial metabolism and oxidative phosphorylation. For ATP production and mitochondrial activation, Pi should be delivered from the cytosol via mitochondrial Pi transporters, such as phosphate carrier (PiC) [38], dicarboxylate carrier (DIC) [39], Mg-ATP/Pi transporter [40], and uncoupling protein-2 (UCP2) [41]. All these transporters are called mitochondrial carriers, which are a superfamily of nuclear-encoded proteins (solute carrier family 25; SLC25) located in the inner mitochondrial membrane [42]. The expression of these mitochondrial carriers varies among cells/tissues, and each of them possess specific characteristics (Figure 2); however, the pathophysiological role of mitochondrial Pi transporters in vascular calcification has not been investigated yet. 

Extramitochondrial Pi has been known to increase mitochondrial membrane potential and superoxide generation in different types of cells, including VSMCs. Pharmacological inhibition of mitochondrial phosphate transporters using butylmalonate (BMA) or mersalyl attenuated mitochondrial hyperpolarization and superoxide production in insulin-secreting cells. Therefore, mitochondrial Pi transporters can be used as therapeutic targets to prevent or relieve high Pi-induced and oxidative stress-mediated osteogenic trans-differentiation and calcification. However, the pathophysiological role of mitochondrial Pi transporters in vascular calcification has not been investigated. Herein, we have introduced the molecular and functional properties of mitochondrial Pi transporters in tissues other than VSMCs.

### 3.1. PiC

PiC (SLC25A3) is encoded at chromosome 12q23 and exists as two isoforms, PiC-A and PiC-B, which are generated via alternative splicing of the exon 3 of *SLC25A3* [43]. While isoform A is mainly found in the heart and skeletal muscle, low levels of isoform B is expressed in all tissues [44]. PiC has six transmembrane domains, and its N and C termini are present within the matrix [43], which is similar to the structure of other mitochondrial carrier proteins, such as ANT and UCP1 [42,45]. PiC is known to be the main route of Pi uptake into the mitochondria, which mediates electroneutral co-transport of Pi and H^+^; thus, Pi transport via PiC might be strictly driven by the pH gradient across the inner mitochondrial membrane [46]. It has been reported that the proximity of PiC with ANT and ATP synthase triggers the composition of the ATP synthasome in the mitochondrial cristae (Ko et al., 2003). This structure could be an important microdomain for ATP synthesis since ADP influx via ANT and Pi uptake via PiC are required for the generation of ATP using ATP synthase.

A recent report showed that PiC can transport not only Pi and H^+^ but also Cu^2+^, which is required for cytochrome c oxidase activity (COX). Indeed, deletion of *SLC25A3* resulted in isolated COX deficiency in murine and human cell lines, which was rescued by the addition of Cu^2+^ into the cell culture medium [47].

Fonyo and Ligeti suggested that PiC may be an important H^+^ donor for the mitochondrial respiratory chain [48]. The role of PiC in ATP production and oxidative phosphorylation has been confirmed in studies using mice models of PiC depletion and loss-of-function mutation in human *SLC25A3*. Baines and Molkentin showed that repression of cardiac muscle-specific PiC exhibited cardiac hypertrophy and slightly reduced fractional shortening [49,50]. In these mice models, mitochondrial Pi uptake and ATP levels decreased, whereas the expression of electron transport chain (ETC) proteins, mitochondrial respiration, and Ca^2+^ homeostasis were not significantly altered. In contrast, PiC expression increased 5-fold in cardiac-specific PiC transgenic mouse without apparent changes in protein levels of ANT, cyclophilin D and ATP synthase, as well as cardiac size and functions [49]. Additionally, PiC upregulation did not affect mitochondrial respiration in mitochondria isolated from cardiac-specific PiC transgenic mice [49]. The above findings regarding overexpression or suppression of PiC highlighted the lack of solid phenotypes, which indicated that the abundance of PiC does not limit the transport of Pi or other potential functions of PiC in the mouse heart. This is consistent with the reports that PiC has an exceptionally high turnover rate [51].

Studies on patients with *SLC25A3* mutations, mainly PiC-A, revealed predominant involvement of cardiac and skeletal muscles, including hypertrophic cardiomyopathy and muscular dystonia [51,52,53]. This preference of the clinical phenotype can be explained by tissue-specific isoforms formed via alternative splicing. Compared to the features of other mitochondrial disorders causing cardiomyopathy, lack of neurocognitive involvement is one of the remarkable features of patients with *SLC25A3* mutations [54].

The mitochondrial PT pore has been suggested to be a multi-protein complex composed of porin, ANT, and cyclophilin D [55]. Halestrap’s group suggested that PiC is the essential component of the mitochondrial PT pore by interacting with cyclophilin D and ANT. Ca^2+^-triggered conformational change of the PiC, facilitated by cyclophilin D, induces pore opening [56]. They also showed that Pi activates MPTP opening in energized and de-energized mitochondria [57]. In the absence of Pi, the sensitivity of PTP to Ca^2+^ or oxidative stress was not affected by the ablation of cyclophilin D [58]. However, the role of mitochondrial Pi transporters on PT pore opening remains controversial.

PiC was also found to functionally interact with the viral mitochondria-localized inhibitor of apoptosis (vMIA) [59]. vMIA inhibits apoptosis by recruiting Bax to mitochondria, thereby inducing its inactivation [60]. Furthermore, vMIA induces mitochondrial fragmentation, which requires PiC [61]. In addition, vMIA decreases mitochondrial respiration and ATP synthesis without changes in the abundance or activity of the F_1_F_0_ ATP synthase or ANT. Despite the lack of any change in the abundance of PiC, vMIA suppresses PiC-mediated mitochondrial Pi uptake and Pi-stimulated ATP synthesis of host cells. Thus, the effect of viral infection on mitochondrial metabolism might be mediated via PiC [59].

In pancreatic β cells, downregulation of PiC triggered reduction in mitochondrial ATP production, leading to attenuation of cytosolic Ca^2+^ oscillation and impaired glucose-stimulated insulin secretion (GSIS) [62]. However, in insulin-secreting clonal cells (INS-1E), PiC suppression did not affect mitochondrial membrane potential, ATP production, and insulin secretion [4]. Further experiments are required to elucidate the contribution of different mitochondrial Pi transport systems to cellular metabolism and bioenergetics.

### 3.2. DIC

DIC is a mitochondrial integral membrane protein encoded by *SLC25A10* in humans, which transports electroneutrally dicarboxylates such as malate, malonate, and succinate in exchange for phosphate, sulfate, and thiosulfate [39,63]. Notably, DIC is expressed most strongly in the liver, kidney, and white adipose tissue and at low levels in other tissues [64,65,66]. The reason behind the differential expression of DIC in various tissues is not clear, which may be related to the functions of those tissues. Remarkably, DIC expression is high in cancer tissues, which might be required for the regulation of oxidative stress and tumor growth. Furthermore, exposure to repetitive hypoxia upregulates DIC, which is involved in cancer cell survival and growth [67]. DIC facilitated not only dicarboxylate-Pi exchange but also exchange of dicarboxylate for dicarboxylate, which was proposed by Chappell and Haarhoff in 1966 [68]. Indeed, the principal function of DIC is to transport dicarboxylates from the cytoplasm into the mitochondria in exchange for Pi. However, along with 2-oxoglutarate carriers, it also catalyzes the uptake of glutathione (GSH) into the matrix by checking the effect of BMA, inhibiting > 80% of GSH efflux [69]. GSH is critical for numerous mitochondrial functions, including membrane structure and integrity, ion homeostasis, and mitochondrial redox status. These findings shed light on the role of DIC in mitochondria. DIC has been proposed to be essential for preserving redox control and normal respiration in rat brain mitochondria. DIC inhibition, resulting in depletion of the mitochondrial GSH pool, led to marked increase in mitochondrial ROS, followed by hyperpolarization of mitochondrial membrane potential and increase in basal respiration rates. Besides that, DIC suppression impaired complex I activity, but not those for complexes II and III [70]. Suppression of DIC expression in clonal insulin-secreting 832/13 cells and isolated rat islets led to inhibition of GSIS by 5–69% [71].

Patients with heterozygous mutation in *SLC25A10* showed severe neurodegenerative disorder, characterized by epileptic encephalopathy, complex I deficiency, and mitochondrial DNA depletion in skeletal muscle [72]. These phenotypes are not related to the functions of DIC, but to decrease in mitochondrial transport of reducing equivalents, anaplerotic Krebs cycle intermediates, or glutathione, which may lead to oxidative stress and mitochondrial dysfunction related to the symptoms observed.

### 3.3. UCP2

Uncoupling protein (UCP) family plays important roles in thermogenesis [73], obesity, diabetes [74], and oxidative stress-related diseases [75]. UCP1 was discovered firstly among members of this family, and UCP2 is a UCP1 ortholog also identified in mitochondria [76]. UCP2 is encoded by *SLC25A8* gene and its mRNA was widely detected in a variety of tissues and cell types [77]. However, at protein level, UCP2 is mainly found in spleen, thymus, bone marrow, pancreas, stomach and lung [78,79]. It has been shown that UCP2 can bind fatty acids (FAs) laterally through its peripheral site, and this binding is critical for UCP2-catalyzed FA flipping across the membrane, which in turn is essential for proton exchange [80], however long-chain FAs are not the only activators of protein transport in UCP2. Additionally, UCP2 has been reported to catalyze the exchange of intramitochondrial C_4_ intermediates, such as malate, oxaloacetate, and aspartate, for cytosolic Pi via a H^+^-supported mechanism for Pi [41]. This electrogenic transport is driven by mitochondrial membrane potential (negative inside) and pH gradient (acidic outside) and is inhibited by the replacement of Gly-268 with Ala in UCP2 [41]. This study examined the physiological role of UCP2 by assessing the change in the levels of C_4_ intermediates, but not that of Pi, as UCP2 might play a minor role in maintaining the mitochondrial Pi pool.

ROS or their byproducts is known to activate UCP2 [81]. Under oxidative stress condition, UCP2 induces proton leak of respiratory chain and decreases mitochondrial ROS production. Therefore, UCP2 can provide a negative feedback loop not to induce overproduction of mitochondrial superoxide. In addition, acute stimulation of UCP2 by ROS directly moderates the glutathionylation status of the UCP to decrease ROS production [82,83]. Mitochondrial ROS production is increased in UCP2 knockout mice, which disturbed mitochondrial dynamic balance towards fission and early damage to mitochondrial ultrastructures upon ischemic stress [84].

### 3.4. Mg-ATP/Pi Carrier

Mitochondrial Mg-ATP/Pi carriers are encoded by *SLC25A23*, *SLC25A24* and *SLC25A25* [40], which transport Pi and adenine nucleotides including ATP, ATP-Mg^2−^, ADP, and AMP [85]. These carriers catalyze electroneutral exchange of divalent phosphate (HPO_4_^2−^) with divalent ATP-Mg (ATP-Mg^2−^) or divalent protonated ADP (H-ADP^2−^) [40,86]. Depending on the concentration gradients of adenine nucleotides and Pi across the inner mitochondrial membrane, ATP-Mg^2−^ or H-ADP^2−^ is incorporated and retained in the mitochondria. The pH gradient is the driving force for Pi accumulation via PiC, which subsequently drives ATP-Mg^2−^ or H-ADP^2−^ accumulation in the mitochondrial matrix. The same mechanism may hold for DIC, which facilitates the mitochondrial transport of malate or other dicarboxylates driven by the Pi gradient generated by PiC. Supporting evidence has shown that ATP-Mg^2−^ uptake or efflux depends on the pH gradient, which is abolished with nigericin and uncouplers [87].

An autosomal dominant mutation in *SLC25A24* manifests as aged phenotypes with loose or wrinkled skin, short stature, hypertrichosis, skull deformities, and a characteristic facial appearance of a depressed nasal bridge, low hairline, and microphthalmia [88]. Interestingly, fibroblasts from patients with *SLC25A24* mutation have altered mitochondrial morphology, decreased proliferation rate, oxygen consumption, and mitochondrial ATP content, whereas mitochondrial membrane potential and oxidative stress sensitivity are high [89,90]. These results indirectly indicated that the aged phenotypes associated with *SLC25A24* mutations may be related to mitochondrial dysfunction and bioenergetic crisis, leading to imbalance in the proliferation and differentiation of progenitor cells of skeletal and connective tissues [52].

An essential characteristic of Mg-ATP/Pi carriers is that the transport of Pi and adenine nucleotides increases after activation by cytosolic Ca^2+^, as four Ca^2+^ binding EF-hand motifs are present on the N-terminal regulatory domain located at the external face of the inner mitochondrial membrane [91,92]. This structure allows the mitochondria to convert cellular Ca^2+^ signals into activation of mitochondrial metabolism. Therefore, Mg-ATP/Pi carriers (SLC25A23-25) are classified as short Ca^2+^-binding mitochondrial carrier (SCaMCs), which contains SLC25A41 also [93].

It has been reported that SLC25A23, but not SLC25A24 and SLC25A25, promotes mitochondrial calcium uptake by interacting with the mitochondrial calcium uniporter (MCU) and MICU1 [94]. Genetic suppression of SLC25A23 decreased mitochondrial Ca^2+^ uptake and augmented agonist-stimulated cytosolic Ca^2+^ transients due to attenuated Ca^2+^ clearance. Furthermore, SLC25A23 silencing reduces basal mitochondrial ROS level, oxidant-induced ATP deterioration, and cell death [94]. Mitochondrial Pi transport is essentially critical for mediating mitochondrial Ca^2+^ signal, and conversely, Ca^2+^ regulates the activity of several mitochondrial Pi transporters. Hence, the close interplay between mitochondrial Pi and Ca^2+^ should be considered when analyzing cellular outcomes.

### 3.5. Oxidative Stress Due to Mitochondrial Pi Transport

Mitochondrial superoxide serves as the largest ROS reservoir in most cell types due to the dynamic activity of the electron transport chain. It is known that ROS production is highly intensified upon mitochondrial hyperpolarization and that only a small increase in the membrane potential can accelerate a large amount of ROS production in the mitochondria [95]. Pi is taken up into mitochondria through the above-described transporters, which may be electrogenic or electroneutral depending on the transport characteristics. Electroneutral transport also can modify mitochondrial membrane potential by altering electrochemical gradients and respiratory chain activity. Studies have shown that high extracellular Pi hyperpolarizes the mitochondrial membrane potential, which is followed by increase in mitochondrial ROS generation and cytotoxic detrimental changes [32,96]. Mitochondrial superoxide generation by high Pi was abolished by collapsing the mitochondrial membrane potential, indicating the critical role of hyperpolarization in ROS production [96].

As the main route of mitochondrial uptake, Pi transport via PiC is driven by the pH gradient across the inner mitochondrial membrane. This subsequently decreases the mitochondrial pH gradient, as Pi transport via PiC is coupled with H^+^ uptake [4]. At the same time, the dissipation of the chemical (pH) gradient can increase the electrical gradient (mitochondrial hyperpolarization) under the maintained proton motive force. Furthermore, Pi significantly accelerates oxidative phosphorylation by activating metabolic enzymes and respiratory chain activity, which additionally exalts the mitochondrial electrical gradient [97]. The further negative mitochondrial membrane potential augments superoxide generation from oxygen by binding with electrons that escaped from the ETC due to higher resistance of proton pumping against the established electrical gradient. This may partially explain how hyperpolarizing response via mitochondrial Pi transporters plays a substantial role in high Pi-induced oxidative stress.

In addition, ROS generation linked to the reverse electron transport (RET) also significantly contributes to the mitochondrial ROS pool. In general, an electron is transferred among respiratory complexes, which finally interacts with O_2_ to form H_2_O. However, about 0.2–2% of the electrons in the ETC do not follow the normal transfer flow and instead directly leak out of the ETC, particularly from complexes I and III, and react with O_2_ to generate superoxide or hydrogen peroxide [98,99]. In this RET, the electrons do not move forward, but some from ubiquinol are transferred back to complex I, reducing NAD^+^ to NADH [100]. A high ratio of ubiquinol to ubiquinone and a high proton motive force are critical for RET [101]. Both rotenone and FCCP remarkably decrease ROS generation via RET [102].

The amount of Pi transported into the mitochondria is remarkably altered by the cytosolic pH; alkaline cytosolic condition increases mitochondrial Pi uptake [27]. Intriguingly, high extracellular Pi can alkalinize cytosolic and mitochondrial pH, which may be related to the buffering action of HPO_4_^2−^/H_2_PO_4_^−^ in the cytosol. Cytosolic alkalinization may accelerate mitochondrial Pi uptake, which augments mitochondrial superoxide generation [27]. Furthermore, cytosolic alkalinization stabilizes the semiquinone radical, a potential superoxide producer, consequently increasing the rate of free radical generation [103]. In addition, mitochondrial matrix alkalinization itself facilitates the opening of the mitochondrial PT pore, resulting in functional impairment [27]. With the Pi-induced changes in cytosolic and mitochondrial pH, extracellular Pi can generate more oxidative stress in the mitochondria and aggravate pathogenic changes.

## 4. Oxidative Stress Related to Cytosolic and Mitochondrial Ca^2+^ and Pi Overloads

In the pathogenic process of vascular calcification, oxidative stress plays a critical role, which is related to upregulation of osteogenic genes and changes in calcification [104,105,106]. As described above, cellular Pi uptake via plasmalemmal and mitochondrial Pi transporters induces ROS generation in a high Pi environment in different types of cells, including VSMCs. Using cytosolic and/or mitochondrial antioxidants, suppression of oxidative stress has been shown to effectively attenuate Pi-induced vascular calcification [32,104]. Cellular Ca^2+^ and Pi synergistically act on the redox system, which later triggers ROS accumulation and oxidative stress. Intracellular Ca^2+^ is disturbed under high Pi conditions, which is associated with the development of many pathological changes. Notably, cellular uptake of Pi influences cytosolic and mitochondrial Ca^2+^ uptake and accumulation.

As described above, high Pi activates depolarization-triggered Ca^2+^ influx via VGCCs in VSMCs [32]. It has been proposed that high levels of Pi activate store-operated Ca^2+^ entry and impair Ca^2+^ efflux from the cytosol in VSMCs [107], leading to the accumulation of cytosolic and mitochondrial Ca^2+^. Interaction between ROS and Ca^2+^ is considered bidirectional, as Ca^2+^ is pivotal for ROS generation, while ROS can control cellular Ca^2+^ signaling [108]. As another consequence of the increase in [Ca^2+^]_i_, the level of Ca^2+^ in the mitochondrial matrix increases due to more Ca^2+^ uptake from the cytosol. This elevation in matrix Ca^2+^ level stimulates the key mitochondrial enzymes of the tricarboxylic acid cycle, including pyruvate dehydrogenase, α-ketoglutarate dehydrogenase, isocitrate dehydrogenase [109,110], which in turn generate more NADH and FADH_2_ and induces excessive ETC activation, subsequently accelerating mitochondrial superoxide generation from ETCs. Moreover, Ca^2+^ activates ATP synthase (complex V) [111,112], α-glycerophosphate dehydrogenase [113], and ANT [114], which enhance ETC activity. Ca^2+^ accumulation promotes oxidative stress by activating nitric oxide synthase (NOS), which blocks complex IV by synthesizing NO, leading to excess ROS formation [115,116,117]. Additionally, Pi reduces the expression of coenzyme Q, while maintenance of this coenzyme in an oxidized state counteracts H_2_O_2_ production and mitochondrial swelling [118].

Mitochondrial Ca^2+^ uptake via MCU is driven by an electrical gradient [119]; thus, ETC activation and hyperpolarization further augments mitochondrial Ca^2+^ uptake. This process is also accelerated by the presence of anions, such as Pi, acetate, β−hydroxybutyrate, glutamate, and bicarbonate, which can provide a H^+^ source for the H^+^ pumps of the respiratory chain. In the absence of adequate Pi, mitochondrial Ca^2+^ uptake, mitochondrial membrane potential, and O_2_ consumption rates are suppressed [120]. Moreover, mitochondrial Pi assists in the formation of insoluble Ca^2+^-Pi salts in the matrix [121] and keeps the low level of ionized mitochondrial Ca^2+^, thereby maintaining active mitochondrial Ca^2+^ uptake by decreasing the uphill limitation of the Ca^2+^ gradient [122,123]. Thus, mitochondrial Pi uptake is possibly an important factor that regulates matrix Ca^2+^ levels and oxidative stress generation by prolonged Ca^2+^ and Pi overload.

**Figure 3:**
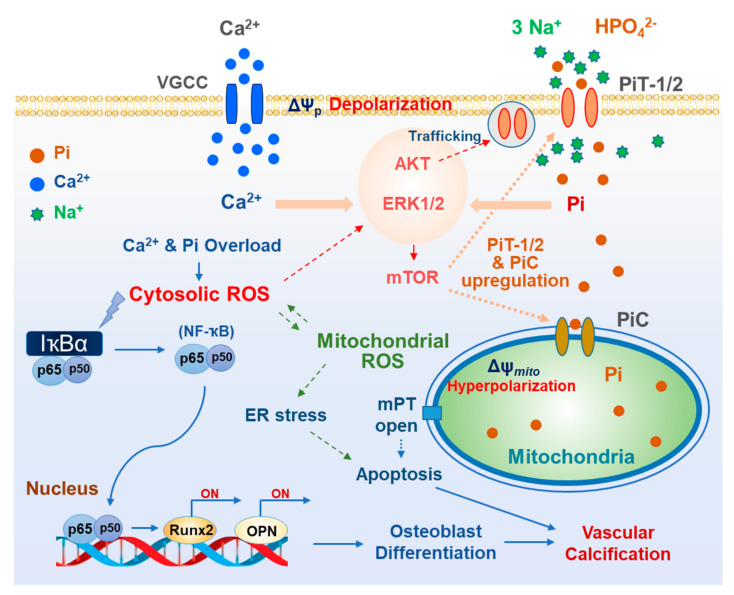
Oxidative stress related to plasmalemmal and mitochondrial Pi transporters in vascular calcification. Type III Na-Pi cotransporters, PiT-1 and PiT-2, can trigger plasma membrane depolarization due to their electrogenic properties. This depolarization couples cytosolic Ca^2+^ influx with Pi, leading to more Ca^2+^ and Pi loading in cytosol. Suppressing the entrance of either Ca^2+^ or Pi successfully attenuate oxidative stress. In addition, Pi influx activates ERK and AKT signaling further augment PiT-1/-2 abundance either by translational regulation or by surface trafficking. Mitochondrial Pi transport, mainly mediated by PiC, elicits mitochondrial hyperpolarization and superoxide generation. All these cytosolic and mitochondrial oxidative stress induces NF-B activation, osteogenic gene upregulation, ER stress and apoptosis leading to vascular calcification.

Together with cytosolic Ca^2+^, the oxidative stress generated by excess Pi activates ERK/mTOR/p70S6K signaling. In turn, mTORC1 potentially suppresses the expression of antioxidant genes such as *CAT*, *SOD1*, and *SOD2*, causing an increase in oxidative stress [124,125]. It is noteworthy that superoxide production by high Pi was effectively inhibited by either decreasing extracellular Ca^2+^ concentration or by chelating intracellular Ca^2+^ [32]. Attenuation of cellular Ca^2+^ load using voltage-gated Ca^2+^ channel blockers also significantly alleviated ROS generation and calcific changes in VSMCs [32]. This indicates the close connection between cellular Pi and Ca^2+^ in oxidative stress-triggered vascular calcification (Figure 3).

## 5. Oxidative Stress and Vascular Medial Calcification

VSMCs of different origins have been used to study the molecular mechanism underlying medial calcification, as they are in the majority in the medial layer of blood vessels. These cells are critical for the contraction and relaxation of blood vessels. Indeed, in most calcifying cell models, short- or long-term incubation with high Pi induces ROS production [2,104,126]. Regarding oxidative stress in calcification, increase in ROS generation is commonly detected in the blood vessels with vitamin D-induced aortic calcification and in rodents with 5/6 nephrectomy on high Pi diet [127,128,129]. In bovine aortic smooth muscle cells treated with β-glycerolphosphate, the mitochondrial ROS released into the cytosol promotes IKKβ phosphorylation, IκBα degradation, and NF-κB nuclear translocation [126]. This NF-κB then acts in the nucleus as a transcription factor to upregulate osteogenic genes such as MSX2 and RUNX2, as well as its downstream ALP. Downregulation of endogenous NF-κB significantly decreases Pi-induced extracellular matrix calcification, suggesting the central role of NF-κB signaling on vascular calcification [126]. Yoshida et al. also demonstrated the smooth muscle cell-selective suppression of NF-κB signaling or reduction in arterial medial calcification in mice with chronic kidney disease (CKD) fed high Pi diet after application of different NF-κB inhibitors [130].

MAPK signaling has been recognized as one of the central players in high Pi-induced calcification in VSMCs and in both human and animal calcification models. Treatment of mouse VSMCs with high Pi causes osteogenic/chondrogenic differentiation in conjunction with amplified phosphorylation of ERK1/2. Application of the MAPK inhibitor, UO126, or knockdown of plasma Pi transporters, PiT-1/-2, prevented the upregulation of RUNX2 upon incubation in the presence of high Pi [131,132]. Indeed, mice with germline ablation of ERK1 and a conditional obstruction of ERK2 in limb mesenchyme (ERK1-/-ERK2Prx1 mice), including osteoblasts, exhibited considerably decreased bone mineralization, indicating the significance of ERK1/2 in osteoblast mineralization [133]. The downstream effects of ERK1/2 during vascular calcification are still not completely understood. However, further studies have shown that ERK1/2 modulates RUNX2 phosphorylation directly at four sites, S43, S301, S319 and S510, among which S301 and S319 contribute to RUNX2 transcriptional activity [134,135]. Recent studies have shown that ERK1/2 repression attenuates calcification by promoting the miR126-3p-DKK/LRP6 pathway [136,137]. This miRNA appears to play a protective role in vascular calcification [138,139]. Oxidative stress elicited by excess Pi is responsible for ERK1/2 activation, as Pi-induced ERK1/2 phosphorylation is abolished by lowering the levels of mitochondrial ROS [32].

The phosphoinositide 3-kinase (PI3K)/AKT pathway also contributes considerably to high Pi-induced increase in VSMC osteo-induction [140,141,142]. Whether activation or inhibition of AKT signaling triggers calcification is still not clear. Deng et al. suggested that AKT phosphorylation is highly critical for RUNX2 ubiquitination and its stabilization [140]. Cellular Pi uptake via PiT-1 was increased by plasma membrane trafficking of PiT-1 after PI3K/AKT activation [32]. In addition, upregulation of AKT/FOXO signaling has been observed in the vasculature of an aging rat model of atherosclerosis [143]. Cui et al. have shown that cell apoptosis and calcification is a result of PI3K/AKT inhibition in rat VSMCs [141]. In addition, FTI-277, a farnesyl transferase inhibitor, can suppress vascular calcification by upregulating PI3K/AKT signaling and preventing apoptosis [142]. A recent study showed that miR155 deficiency attenuated, whereas miR155 overexpression induced by high levels of Ca^2+^ and Pi enhanced vascular calcification by regulating AKT activation and FOXO3a degradation [144]. Indeed, mitochondrial ROS aggravates PTEN oxidative inactivation, which is crucial for inducing PI3K/AKT/mTOR signaling [145].

A recent work has been shown that ER stress intensified the calcification of VSMCs by stimulating the release of Grp78-loaded extracellular vesicles [146]. Additionally, activated mTORC1 potentiates ER stress, resulting in diminished production of an endogenous inhibitor of mineralization and osteoblastic trans-differentiation of VSMCs in CKD mice [147]. It is noteworthy that mitochondria and ER have a tight connection, because of which mitochondrial dysfunction induces ER stress and vice versa [148]. Indeed, high Pi level promotes mitochondrial ROS production in insulin-secreting cells, eliciting ER stress and apoptosis, which were prevented by a mitochondrial superoxide scavenger [96]. Vascular calcification is accompanied by upregulation of ER stress markers, including GRP78, GRP94 and CHOP, and consequent apoptosis in VSMCs [149]. The PERK-eIF2α-ATF4-CHOP complex is formed as a part of the integrated stress response and it is upregulated in animal models of vascular calcification [150,151]. In human VSMCs, oxidative stress induces ER stress via the IRE-1-XBP1-GRP78 pathway. In particular, XBP-1, upregulated by ER stress, can bind to the *RUNX2* promoter, eliciting VSMC differentiation and calcification [152]. More clarification regarding the connection between mitochondrial ROS and ER stress is required to understand the mechanism underlying progression of vascular calcification.

## 6. Conclusions

**Figure 4:**
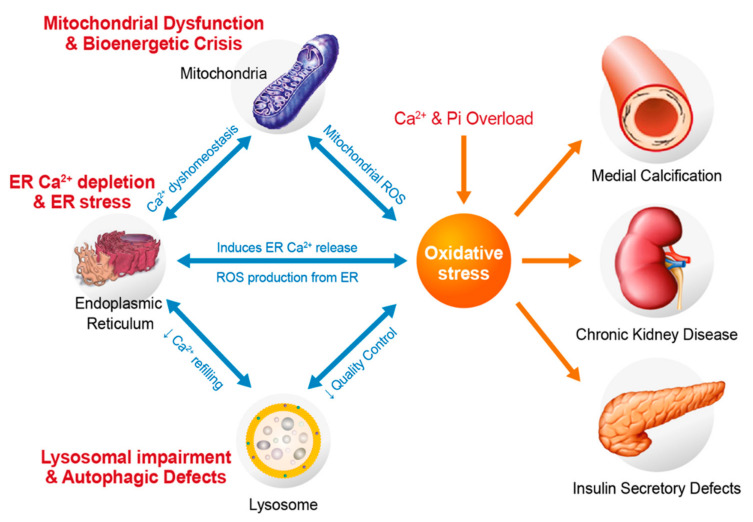
Oxidative stress by Ca and Pi overloads in vascular calcification. Ca and Pi overloads accelerates cytosolic and mitochondrial oxidative stress, which can induce more mitochondrial dysfunction and ER stress. Depletion of the ER Ca pool further increases ROS generation from mitochondrial and the ER, which also disturbs lysosomal Ca homeostasis and autophagy defects. All these pathologic alterations in organelles aggravate oxidative stress and disease progression including arterial medial calcification.

As described above, regulation of Pi uptake via plasmalemmal and mitochondrial Pi transporters is related to the pathophysiology of oxidative stress-induced vascular calcification. Plasmalemmal Pi transport can induce cytosolic Ca^2+^ accumulation due to depolarization-activated Ca^2+^ influx. Mitochondrial Pi transport also increases net Ca^2+^ uptake due to augmented electrical driving force and free Ca^2+^ gradients. Oxidative stress due to Ca^2+^ and Pi overload induces mitochondrial dysfunction, ER stress, and lysosomal dysfunction, all of which are caused or aggravated by perturbations in Ca^2+^ homeostasis (Figure 4). Excessive intracellular and intramitochondrial Ca^2+^ and Pi synergistically increase the levels of cytosolic and mitochondrial ROS. Thus, suppressing either Ca^2+^ or Pi overload may abrogate pathological oxidative stress and disease progression.

It is noteworthy that elevated Pi levels initiate a positive feedback activation loop, establishing a vicious cycle of Pi toxicity. Pi uptake mediated by PiT-1 increases the abundance of functional PiT-1 by translational upregulation and surface trafficking. Our recent unpublished data showed that high Pi treatment increased the abundance of mitochondrial PiC in an ERK1/2-mTOR-dependent manner (data not shown). Cytosolic alkalinization due to Pi uptake also accelerates mitochondrial Pi influx and facilitates PT pore opening. All these mechanisms amplify Pi overload-induced detrimental changes in the cytosol and mitochondria after oxidative stress. On the contrary, this positive feedback mechanism might prevent disease progression by inhibiting any one component of the signal amplification loop. Further investigations to elucidate the mechanism via which Pi accumulates with Ca^2+^ and generates oxidative stress, and its pathological consequences, may reveal a novel therapeutic strategy against vascular calcification.
